# The inhibition of IL-2/IL-2R gives rise to CD8^+^ T cell and lymphocyte decrease through JAK1-STAT5 in critical patients with COVID-19 pneumonia

**DOI:** 10.1038/s41419-020-2636-4

**Published:** 2020-06-08

**Authors:** Hongbo Shi, Wenjing Wang, Jiming Yin, Yabo Ouyang, Lijun Pang, Yingmei Feng, Luxin Qiao, Xianghua Guo, Honglin Shi, Ronghua Jin, Dexi Chen

**Affiliations:** 1 Beijing Institute of Hepatology, Beijing Youan Hospital, Capital Medical University, 100069 Beijing, China; 2 Beijing Engineering Research Center for Precision Medicine and Transformation of Hepatitis and Liver Cancer, Beijing, China

**Keywords:** Interleukins, Viral infection

## Abstract

Although most patients with COVID-19 pneumonia have a good prognosis, some patients develop to severe or critical illness, and the mortality of critical cases is up to 61.5%. However, specific molecular information about immune response in critical patients with COVID-19 is poorly understood. A total of 54 patients were enrolled and divided into three groups, among which 34 were common, 14 were severe, and 6 were critical. The constitution of peripheral blood mononuclear cells (PBMC) in patients was analyzed by CyTOF. The profile of cytokines was examined in plasma of patients using luminex. The IL-2 signaling pathway was investigated in the PBMC of patients by qRT-PCR. The count and percentage of lymphocytes were significantly decreased in critical patients compared to common and severe patients with COVID-19 pneumonia. The count of T cells, B cells, and NK cells was remarkably decreased in critical patients compared to normal controls. The percentage of CD8^+^ T cells was significantly lower in critical patients than that in common and severe patients with COVID-19 pneumonia. The expression of IL-2R, JAK1, and STAT5 decreased in PBMC of common, severe, and critical patients, but IL-2 level was elevated in severe patients and decreased in critical patients with COVID-19 pneumonia. The decrease of CD8^+^ T cells in critical patients with COVID-19 pneumonia may be related to the IL-2 signaling pathway. The inhibition of IL-2/IL-2R gives rise to CD8^+^ T cell and lymphocyte decrease through JAK1-STAT5 in critical patients with COVID-19 pneumonia.

## Introduction

In early December 2019, cases of unknown pneumonia were found in Wuhan, Hubei province in China, which were subsequently identified as an acute respiratory infectious disease caused by a novel coronavirus^1–3^. On 11 February 2020, the World Health Organization named these diseases as Corona Virus Disease 2019 (COVID-19). This infectious disease is highly contagious and threatens human life and health^4–7^.

The clinical spectrum of COVID-19 pneumonia includes mild, common, severe, and critical cases. Although most patients with COVID-19 pneumonia have a good prognosis, some patients develop to severe or critical illness, and the mortality of critical cases can reach up to 61.5%^8^. However, specific molecular information about immune responses in critical patients with COVID-19 remains poorly understood.

Reduction of mortality in critical patients is crucial for clinical response against COVID 19. In this study, we analyzed the potential molecular mechanism underlying the decrease of lymphocytes in critical patients with COVID-19 pneumonia, which may provide the new therapeutic targets for COVID-19.

## Results

### The decrease of lymphocytes in critical patients with COVID-19 pneumonia

**Fig. 1:**
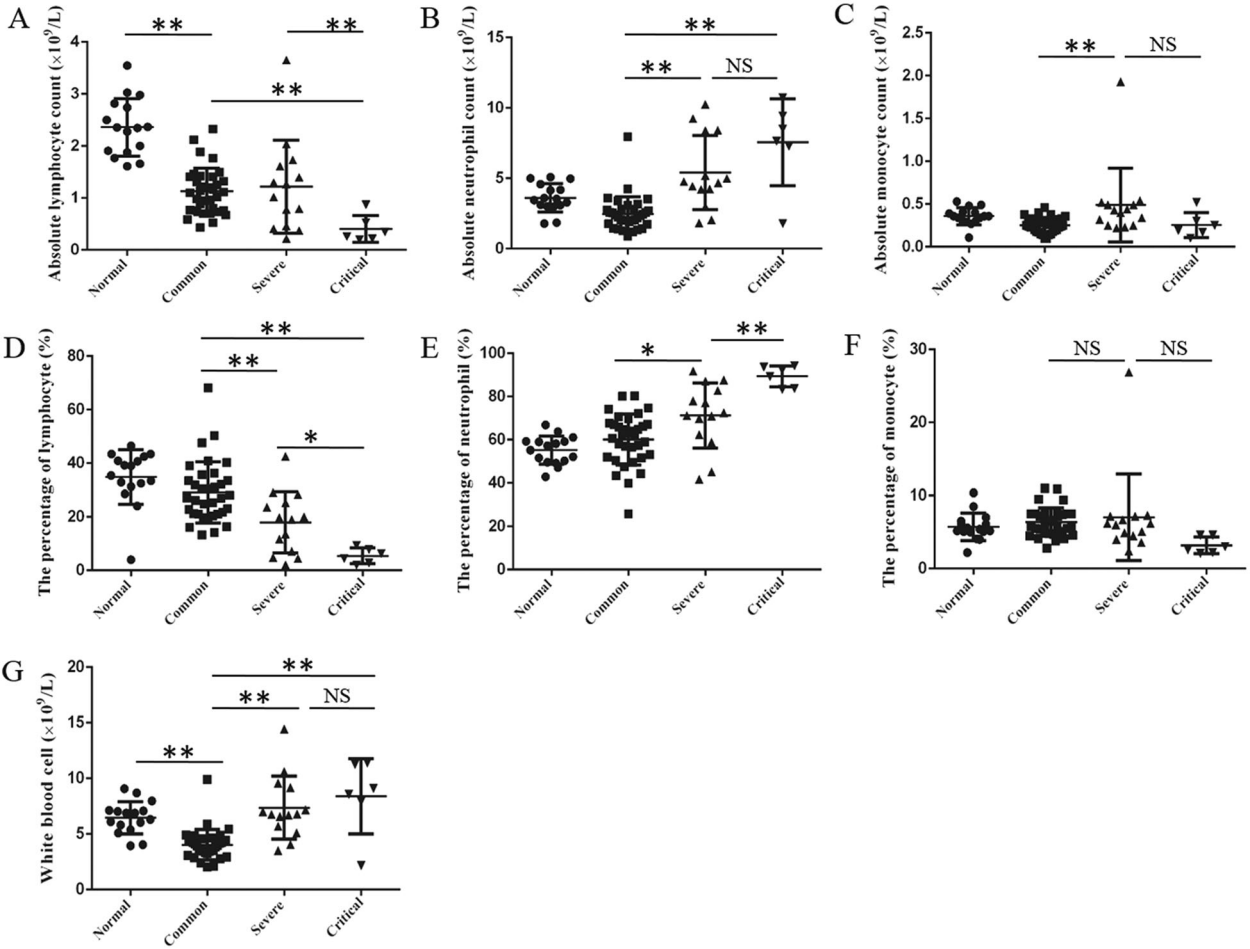
The blood routine analysis in patients with COVID-19 pneumonia. The patients were enrolled and divided into common, severe, and critical types. The absolute count of lymphocyte (**a**), neutrophil (**b**), monocyte (**c**), and the percentage of lymphocyte (**d**), neutrophil (**e**), monocyte (**f**), and white blood cell count (**g**) in patients and normal controls were acquired by full-automatic blood routine detector. One-way analysis of variance (ANOVA) followed by post hoc LSD was used to compare differences between groups. *P < 0.05 and **P < 0.01.

Patients with COVID-19 pneumonia were enrolled and divided into common, severe, and critical type by the guidance of the National Health Commission of China^9^ (Table 1). The absolute lymphocyte count in patients with COVID-19 pneumonia was remarkably decreased compared with normal controls (Fig. 1a). Notably, the absolute lymphocyte count in critical patients was significantly lower than that in common or severe patients, but there was no difference between common patients and severe patients (Fig. 1a). In addition, the percentage of lymphocyte in white blood cells (WBC) decreased in turn in common, severe, and critical patients, and the differences among them were statistically significant (Fig. 1d). Meanwhile, the WBC count increased in turn in common, severe, and critical patients, and there was a statistical difference between common and critical patients (Fig. 1g). The increase in WBC count was driven by an increase in absolute neutrophil count (Fig. 1b), which was consistent with the trend of C reactive protein and procalcitonin (Table 2). All in all, the count and percentage of lymphocyte were decreased in critical patients compared to common and severe patients with COVID-19 pneumonia.

**Table 1:** Clinical categorization of the patients with COVID-19 pneumonia by National Health Commission of China.

Common	Severe	Critical
	Meet any of the followings:	Meet any of the followings:
Fever	1. Respiratory distress, RR ≥30/min	1. Respiratory failure and mechanical ventilation
Respiratory symptoms	2. Saturation of oxygen ≤93% at rest state	2. Shock
Pneumonia on X-ray or CT	3. Arterial partial pressure of oxygen (PaO2) or Fraction of inspiration O2 (FiO2) ≤300 mnHg (1 mmHg = 0.133 kPa)	3. Other organ failure and ICU monitoring and treatment

**Table 2:** The clinical information of normal controls and patients with COVID-19 pneumonia.

	Normal controls (*n* = 16)	Common patients (*n* = 34)	Severe patients (*n* = 14)	Critical patients (*n* = 6)	*P* value
Ages (years old)	55 ± 13	50 ± 15	63 ± 5	75 ± 10	−
Gender(male/female)	6/10	14/20	6/8	2/4	−
White blood cell (×10^9^/L)	6.46 ± 0.36	4.02 ± 0.23	7.36 ± 0.76	8.39 ± 1.38	0.000
Neutrophil (×10^9^/L)	3.61 ± 0.25	2.47 ± 0.21	5.41 ± 0.71	7.57 ± 1.26	0.000
Lymphocyte (×10^9^/L)	2.36 ± 0.14	1.13 ± 0.08	1.22 ± 0.24	0.41 ± 0.11	0.000
Monocyte (×10^9^/L)	0.36 ± 0.02	0.25 ± 0.02	0.49 ± 0.11	0.26 ± 0.06	0.005
Neutrophil (%)	55.18 ± 1.63	60.07 ± 2.02	71.24 ± 4.03	89.38 ± 1.99	0.000
Lymphocyte (%)	34.86 ± 2.56	29.11 ± 1.95	17.98 ± 3.07	5.54 ± 1.18	0.000
Monocyte (%)	5.72 ± 0.47	6.37 ± 0.33	7.01 ± 1.58	3.18 ± 0.47	0.082
Alanine aminotransferase (U/L)	19.85 ± 2.33	49.53 ± 9.8	49.29 ± 9.1	49.17 ± 15.06	0.232
Aspartate aminotransferase (U/L)	22.23 ± 1.71	38.53 ± 5.41	39.14 ± 5.79	85.17 ± 31.05	0.003
Total bilirubin (μmol/L)	14.71 ± 1.29	9.73 ± 0.56	11.29 ± 2.16	18.40 ± 2.24	0.000
Direct bilirubin (μmol/L)	4.67 ± 0.47	1.91 ± 0.12	3.79 ± 1.81	6.52 ± 1.58	0.006
Total protein (g/L)	74.93 ± 1.02	73.52 ± 0.92	71.69 ± 2.02	69.52 ± 3.64	0.241
Albumin (g/L)	46.1 ± 0.54	36.70 ± 0.71	31.05 ± 0.96	30.70 ± 1.88	0.000
Creatinine (μmol/L)	56.19 ± 2.92	59.94 ± 2.76	56.43 ± 3.66	96.83 ± 25.72	0.002
Glomerular filtration rate (mL/min/1.73 m^2^)	126.59 ± 5.62	113.32 ± 5.62	101.19 ± 3.47	70.85 ± 14.15	0.000
Procalcitonin (ng/mL)	0.10 ± 0.001	0.13 ± 0.004	0.18 ± 0.04	3.42 ± 3.15	0.015
C reactive protein (mg/L)	2.50 ± 0.25	16.50 ± 2.73	46.34 ± 9.82	96.73 ± 21.72	0.000
Saturation of oxygen (%)	98.53 ± 1.21	96.83 ± 0.90	92.84 ± 0.59	87.03 ± 2.95	0.019

### The constitution of PBMC in patients with COVID-19 pneumonia

**Fig. 2:**
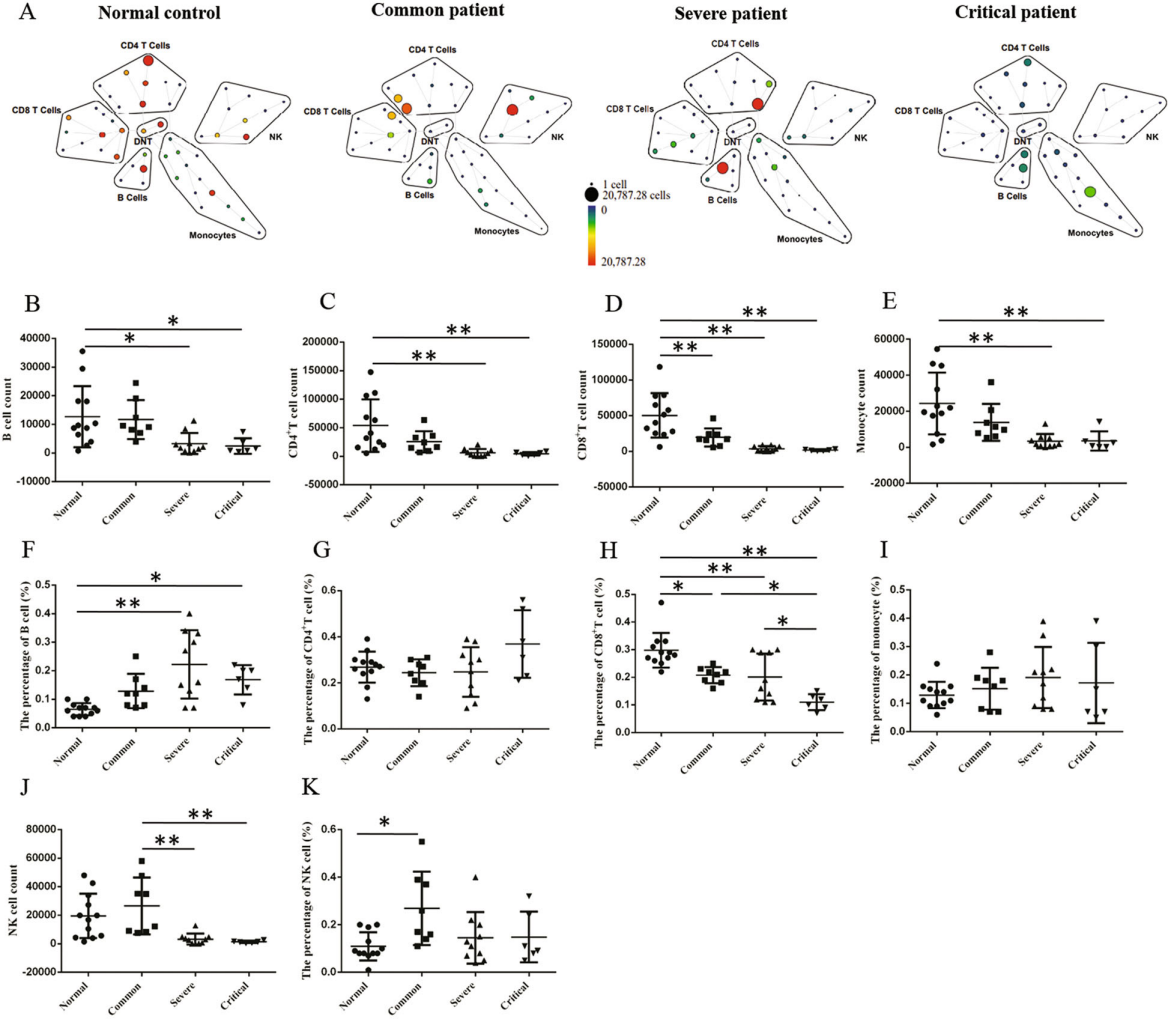
The CyTOF analysis in PBMC of patients with COVID-19 pneumonia. The patients were enrolled and divided into common, severe, and critical types. The count and percentage of B cell (**a**, **b**, **f**), CD4^+^ T cell (**a**, **c**, **g**), CD8^+^ T cell (**a**, **d**, **h**), monocyte (**a**, **e**, **i**), and NK cell (**a**, **j**, **k**) in PBMC of patients and normal controls was analyzed by CyTOF. ANOVA followed by post hoc LSD was used to compare differences between groups. *P < 0.05 and **P < 0.01.

To investigate the cause of lymphocytic decrease, we analyzed the constitution of peripheral blood mononuclear cell (PBMC) in patients with COVID-19 pneumonia by CyTOF. As well as we know, PBMC consists largely of lymphocytes and lymphocyte includes T cell, B cell, and NK cell, and T cell is the main part. CyTOF revealed that the count of T cells, B cells, NK cells, and monocytes was remarkably decreased in critical patients and severe patients compared to normal controls (Fig. 2a–e, j). In addition, the percentage of B cells was increased in severe and critical patients compared to normal controls (Fig. 2f). The percentage of NK cells in common patients was higher than that in normal controls (Fig. 2k). The percentage of CD4^+^ T cells and monocytes had no statistical difference among the patients and normal controls (Fig. 2g, i). However, the percentage of CD8^+^ T cells was significantly lower in patients with COVID-19 pneumonia than that in normal controls, especially the percentage of CD8^+^ T cells in critical patients was decreased compared to common and severe patients with COVID-19 pneumonia (Fig. 2h).

### The profile of cytokines in patients with COVID-19 pneumonia

**Fig. 3:**
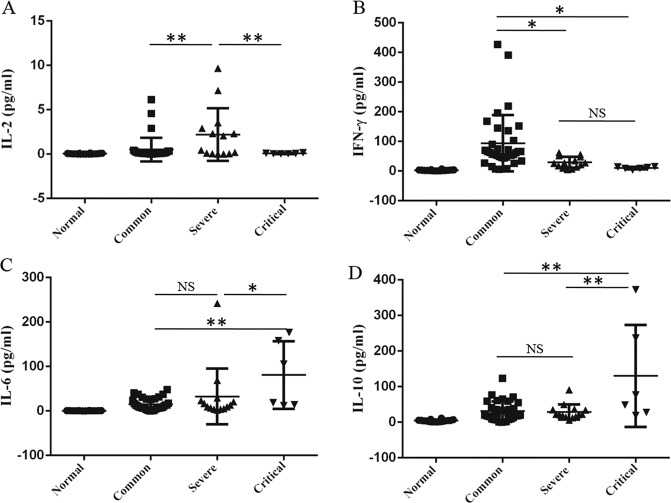
The cytokine profile in plasma of patients with COVID-19 pneumonia. The patients were enrolled and divided into common, severe, and critical types. The levels of II-2 (**a**), IFN-γ (**b**), IL-6 (**c**), and IL-10 (**d**) in plasma of patients and normal controls were analyzed by luminex. The experiment shown was replicated in the laboratory twice. ANOVA followed by post hoc LSD was used to compare differences between groups. *P < 0.05 and **P < 0.01.

To make further investigation, we analyzed the profile of cytokines in plasma of patients with COVID-19 pneumonia using luminex. The level of interleukin-2 (IL-2) in critical patients was significantly lower than that in severe patients, whereas the level of IL-2 in severe patients was higher than that in normal controls and common patients (Fig. 3a). For interferon-γ (IFN-γ), its level in critical patients was significantly decreased compared to common patients, but its level in common patients was increased compared to the normal controls (Fig. 3b). Contrary to IL-2 and IFN-γ, the level of interleukin-6 (IL-6) and interleukin-10 (IL-10) in critical patients was higher than that in common and severe patients with COVID-19 pneumonia (Fig. 3c, d). Because IL-2 is required for T cells proliferation, differentiation, and activation^10^, we speculated that the decrease of CD8^+^ T cells in critical patients with COVID-19 pneumonia might be related to the IL-2 signaling pathway.

### The expression of IL2 receptor, JAK1, STAT5 in patients with COVID-19 pneumonia

**Fig. 4:**
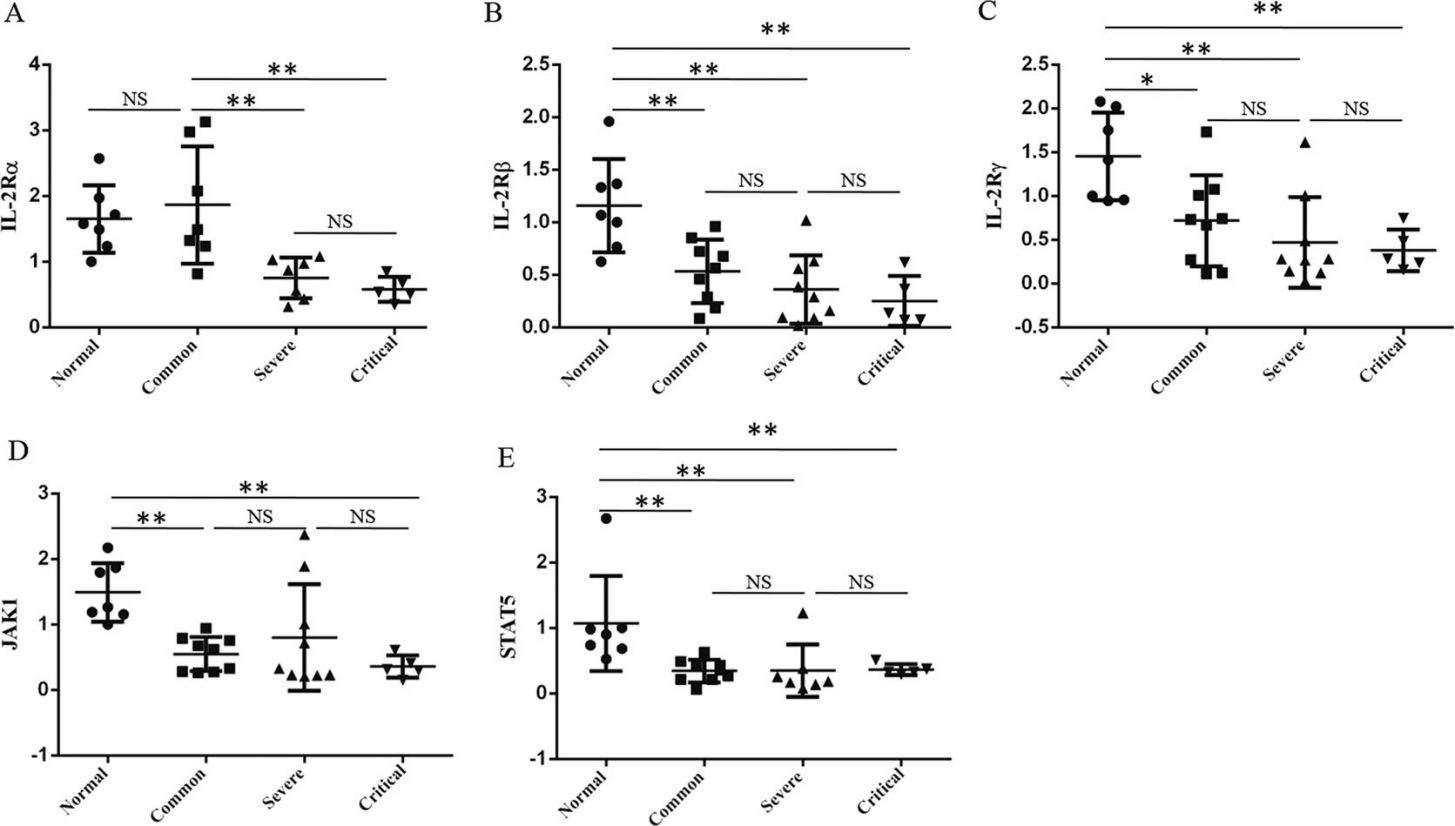
The expression of IL-2 signaling pathway in PBMC of patients with COVID-19 pneumonia. The patients were enrolled and divided into common, severe, and critical types. The expression of IL-2Rα (**a**), IL-2Rβ (**b**), IL-2Rγc (**c**), JAK1 (**d**), and STAT5 (**e**) in PBMC of patients and normal controls were detected by qRT-PCR. The experiment shown was replicated in the laboratory twice. ANOVA followed by post hoc LSD was used to compare differences between groups. *P < 0.05 and **P < 0.01.

It has been reported that IL-2 and IL-2 receptor (IL-2R) provides an important signal for T cell activation through JAK1-STAT5 signaling pathway^11^. IL-2R has three subunits: IL-2Rα, IL-2Rβ, and IL-2Rγc, and the activated T cells can simultaneously express α, β, and γc chain and form a high-affinity receptor^12^. We found that the IL-2Rα expression in PBMC of severe and critical patients was significantly lower than that in common patients and normal controls (Fig. 4a). In addition, the expression of IL-2Rβ and IL-2Rγc in common, severe, and critical patients was significantly lower than that in normal controls, whereas there was no statistical difference among those patients with COVID-19 pneumonia (Fig. 4b, c). Accordingly, the expression of JAK1 and STAT5 showed similar trends as IL-2Rβ and IL-2Rγc in patients and normal controls (Fig. 4d, e). Thus, the decrease of T cells in patients with COVID-19 pneumonia was correlated to low expression of IL-2R and JAK1-STAT5, especially in severe and critical patients.

## Discussion

The most significant finding of this study was the low level of IL-2 in plasma and low expression of IL-2R in PBMC of critical patients, which may result in the remarkable decrease of CD8^+^ T cell and lymphocytes in critical patients with COVID-19 pneumonia. In addition, we found that total T cell, B cell, and NK cell counts were remarkably decreased in critical patients compared to normal controls.

At present, the rising epidemic situation in China has been effectively curbed through a series of preventive control and medical treatment. The new confirmed and suspected cases in China are basically cleared, and the treatment of remaining severe and critical cases has become the most important task. According to the latest guidelines for novel coronavirus pneumonia, the progressive decrease of peripheral blood lymphocytes is one of the clinical early warning indicators for adult patients with severe and critical illness. Similar to previous studies^13^, we also found that peripheral blood lymphocytes were decreased in the patients with COVID-19 pneumonia, especially in critical patients. Importantly, we found that CD8^+^ T cells were remarkably decreased, which was the same as another study^14^. Some studies have also shown that critical patients with COVID-19 pneumonia have an immune deficiency and hypoimmunity, which may also lead to serious infection and death^15^. Therefore, CD8^+^ T cell reduction may result in underlying increased mortality in critical patients.

The secretion of cytokines plays an important role in the development and differentiation of immune cells. Many studies have suggested that cytokine storms might be one of the causes of multiple organ failure and death in severe and critical patients with COVID-19 pneumonia^16,17^. Similarly, our study also found that some inflammatory cytokines such as IL-6 and IL-10 increased in critical patients with COVID-19 pneumonia. Differently, we found that IL-2 was elevated in severe patients but decreased in critical patients with COVID-19 pneumonia. IL-2, also known as the T cell growth factor, is mainly produced by activated CD4^+^ T cells and CD8^+^ T cells^18–21^. It has been demonstrated that IL-2 at high concentration enhances CD4^+^ T and CD8^+^ T cell activation by stimulating expansion and differentiation of conventional T cells and IL-2 at low concentration inhibits CD4^+^ T and CD8^+^ T cell activation by maintaining activity and survival of T regulatory cells (Treg)^21^. IL-2 is so specific and critical for T cells activation that we investigate the IL-2 signaling pathway. Consistent with IL-2 level in plasma, we found that the expression of IL-2R and JAK1-STAT5 in PBMC was decreased in critical patients with COVID-19 pneumonia. Therefore, the decrease of CD8^+^ T cells and lymphocytes in critical patients with COVID-19 pneumonia may be related to the inhibition of IL-2 signaling pathway.

In addition, we also observed that IL-10 and IL-6 was increased and IFN-γ was decreased in critical patients with COVID-19 pneumonia. Relative to IL-2, IL-10, IL-6, and IFN-γ have more versatile functions. IL-10 is considered a prototypical anti-inflammation cytokine^22^, but the recent findings indicate that high exogenous IL-10 level promotes the CD8^+^ T cell cytotoxicity and intermediate endogenous IL-10 level induces exhaustion of CD8^+^ T cells in tumor^23,24^. IL-10 can inhibit IL-2 secretion and has heterogenity on CD8^+^ T cell activation which may depend on other environmental cytokines^24^, so we speculate that it may take part in T cell activation through IL-2 pathway. IL-6 can induce the differentiation of T cells, whereas its level was increased in our study, so we think it plays an important role in secretion of acute phase proteins such as C reactive protein and inflammatory cytokines^25^. IFN-γ has a critical role in recognizing and eliminating pathogens, but it has no direct effects on CD8^+^ T cells activation^26,27^. Therefore, we focus on the IL-2 pathway to explore its possible effect on CD8^+^ T cells and lymphocytes.

This study is limited by sample size for critical patients with COVID-19 pneumonia, and clinical significance and power would be increased with more patients. However, our findings still provide evidences and clues for the diagnosis and treatment of critical patients with COVID-19 pneumonia. The progressive decrease of IL-2 in plasma may be a warning factor of disease deterioration in patients with COVID-19 pneumonia. For critical patients with COVID-19 pneumonia, appropriate IL-2 supplementation could be beneficial by improving the immune disorder so as to reduce mortality.

## Materials and methods

### Patients

All patients were enrolled from inpatients in Beijing Youan Hospital, Capital Medical University. The patients were confirmed by nucleic acid detection of novel coronavirus using the protocol as described previously^6,28^. A total of 54 patients were divided into three groups according to the guidance of the National Health Commission of China^9^ (Table 1), among which 34 were common type, 14 were severe type, and 6 were critical type. Sixteen normal controls were from the physical examination population in Beijing Youan Hospital, Capital Medical University. The clinical information of normal controls and patients with COVID-19 pneumonia was described in Table 2. The informed consent was obtained from all the patients. The study was approved by the Ethics Committee of Beijing Youan Hospital, Capital Medical University, China.

### CyTOF analysis

Peripheral blood mononuclear cells (PBMC) were cultured with 2 μM cisplatin (195-Pt, Fluidigm) for 2 min before quenching with CSB (Fluidigm) to identify viability. A fix I (Fluidigm) buffer was then used to fix cells for 15 min at room temperature (RT). All metal-conjugated antibodies (Fluidigm) were titrated for optimal concentration before staining with cells. Cells were cultured with antibodies in a total 50 μL CSD (Fluidigm) for 30 min at RT, triple washed in CSB, and incubated with 0.125-μm intercalator in fix and perm buffer (Fluidigm) at 4 °C overnight. Prior to the acquisition, samples were resuspended in deionized water containing 10% EQ4 element beads (Fluidigm), and the concentration of cells was adjusted into 1 × 10^6^ cell/ml. Data acquisition was performed on a Helios mass cytometer (Fluidigm). Data analysis was performed according to the previous paper^29,30^.

### Luminex assay

Cytokines in human plasma were analyzed by luminex assay (Millipore, Billerica, USA). The diluted standard and quality control was added into appropriate wells. The assay buffer and sample was added into sample wells. The premixed beads were added into each well. The plate was sealed and incubated in plate shaker overnight at 4 °C. After washing the plate, the detection antibody was added into each well and incubated for 1 h at room temperature. The streptavidin–phycoerythrin was added into each well-containing detection antibody for 30 min at room temperature. After washing, sheath fluid was added into all wells, and the plate was read on FlexMAP3D.

### qRT-PCR

Total RNA was extracted according to the instructions of the RNeasy Micro Kit (Qiagen, CA, USA), and 4 μl of total RNA was reverse transcribed into cDNA by SuperScript III First Strand cDNA synthesis kit (Invitrogen, CA, USA). After diluting the cDNA for 20-fold, quantitative real-time PCR was performed on a high-throughput qRT-PCR instrument (Fluidigm, USA) using 1 μl of cDNA and Applied Biosystems Gene Expression Arrays with Universal Taqman Fast Master Mix (Applied Biosystems, CA, USA). The PCR conditions were 2 min at 50 °C, then 2 min at 95 °C followed by 50 cycles at 95 °C for 15 s, 56 °C for 30 s, and 72 °C for 50 s. A glyceraldehyde 3-phosphate dehydrogenase (GAPDH) housekeeping gene was used as a reference control. The primers and probes of qRT-PCR were seen in Table 3.

**Table 3:** The primer and probe of qRT-PCR.

	Primer and probe 5′−3′
IL2Rβ FORWARD	CGCATCCTTAAGCAGCAACC
IL2Rβ REVERSE	AGGCCTCTATCTCCAAGGCA
IL2Rβ PROBE	TCGCTGACCAGCTGCTTCACCAACCA
IL2Rα FORWARD	TTATCATTTCGTGGTGGGGCA
IL2RαREVERSE	CTTGTCTTCCCGTGGGTCAT
IL2Rα PROBE	ACAGAGGTCCTGCTGAGAGCGTCTGCA
IL2Rγ FORWARD	CCACTGTTTGGAGCACTTGG
IL2Rγ REVERSE	CCGAACACGAAACGTGTAGC
IL2Rγ PROBE	CCGGACTGACTGGGACCACAGCTGG
STAT5A FORWARD	AAGCCCCACTGGAATGATGG
STAT5A REVERSE	GGAGTCAAACTTCCAGGCGA
STAT5A PROBE	AAGCAACAGGCCCACGACCTGCTCA
JAK1 FORWARD	ACGAGTGTCTAGGGATGGCT
JAK1 REVERSE	CGCATCCTGGTGAGAAGGTT
JAK1 PROBE	TGCAGTTGCCAGAACTGCCCAAGGACA
GAPDH FORWARD	CAGCCTCAAGATCATCAGCA
GAPDH REVERSE	TGTGGTCATGAGTCCTTCCA
GAPDH PROBE	CAAGGTCATCCATGACAACTTTGG

### Statistical analysis

All data was analyzed using GraphPad 6.02. One-way analysis of variance (ANOVA) followed by post hoc LSD was used to compare differences between groups. Differences were considered statistically significant at confidence levels *P < 0.05 or **P < 0.01.

## Supplementary information

Figure S1

Supplementary Figure Legends
