# Supplementary figures and images for: The inhibition of IL-2/IL-2R gives rise to CD8+ T cell and lymphocyte decrease through JAK1-STAT5 in critical patients with COVID-19 pneumonia
Source: Cell Death Dis. 2020 Jun 8;11(6):429. doi: 10.1038/s41419-020-2636-4 (PMC7276960; doi:10.1038/s41419-020-2636-4)

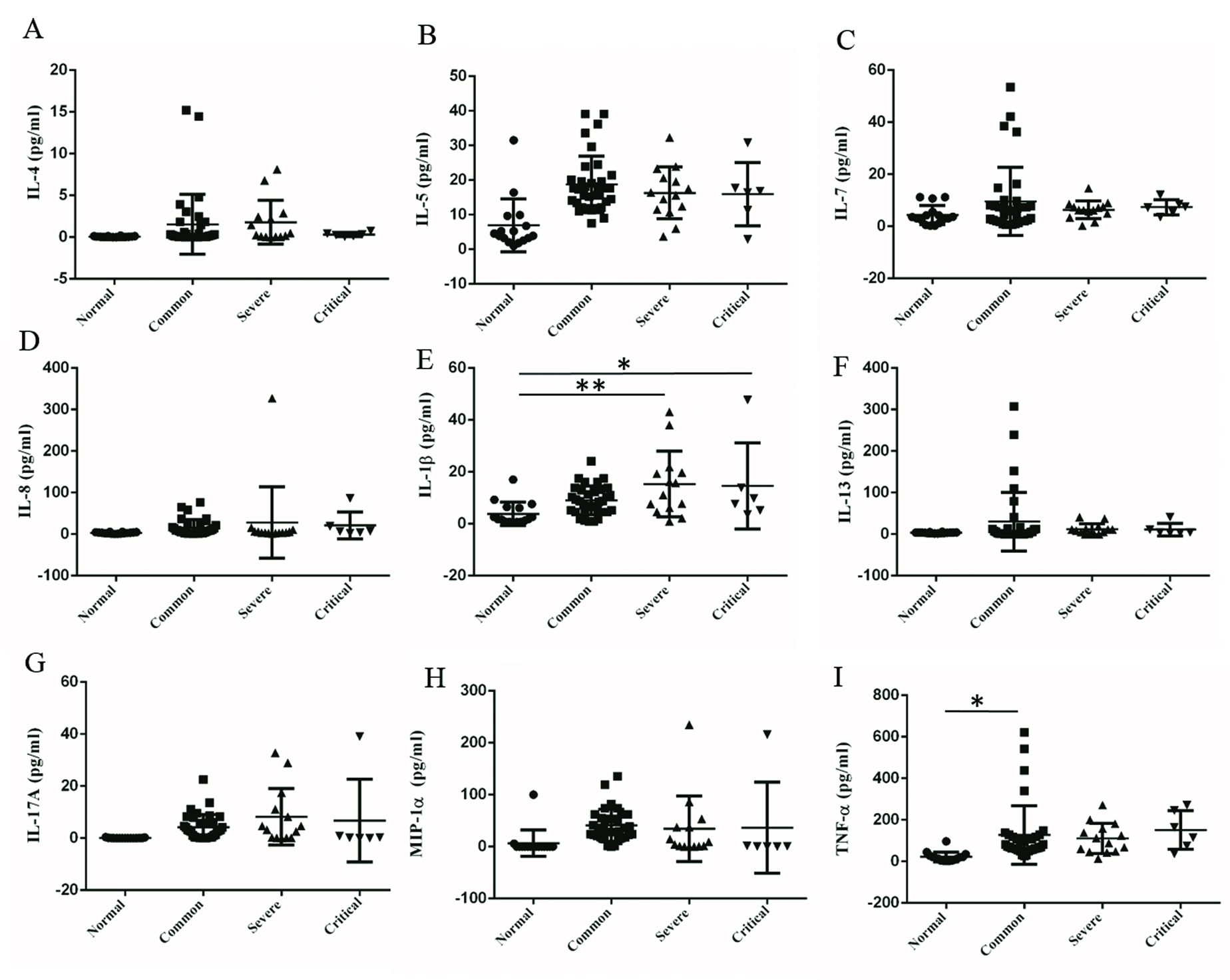

Supplement: Supplementary file 1 — Figure S1 [file 41419_2020_2636_MOESM1_ESM.tif]
