# Supplementary material for: The inhibition of IL-2/IL-2R gives rise to CD8+ T cell and lymphocyte decrease through JAK1-STAT5 in critical patients with COVID-19 pneumonia
Source: Cell Death Dis. 2020 Jun 8;11(6):429. doi: 10.1038/s41419-020-2636-4 (PMC7276960; doi:10.1038/s41419-020-2636-4)
Supplement: Supplementary file 2 — Supplementary Figure Legends [file 41419_2020_2636_MOESM2_ESM.docx]

**Supplementary Figure Legend**

**Figure S1. The cytokine profile in plasma of patients with COVID-19 pneumonia.** The patients were enrolled and divided into common, severe, and critical types. The levels of IL-4 (A), IL-5 (B), IL-7 (C), IL-8 (D), IL-1β (E), IL-13 (F), IL-17A (G), MIP-1α (H) and TNF-α (I) in plasma of patients and normal controls were analyzed by luminex. The experiment shown was replicated in the laboratory twice. ANOVA followed by post hoc LSD was used to compare differences between groups. *indicated P < 0.05 and ** indicated P < 0.01.
